# Prior Entrepreneurial Exposure and Action of Women Entrepreneurs: Exploring the Moderation Effects of Entrepreneurial Competencies in a Developing Country Context

**DOI:** 10.3389/fpsyg.2020.00922

**Published:** 2020-05-26

**Authors:** Melodi Botha

**Affiliations:** Department of Business Management, University of Pretoria, Pretoria, South Africa

**Keywords:** prior entrepreneurial exposure, entrepreneurial action, entrepreneurial competencies, women entrepreneurs, developing country

## Abstract

While the differences between men and women with regard to entrepreneurial activity is well-acknowledged, few scholars have explored models explaining the differences through an objectivist lens. This research addresses this gap by investigating the relationship between prior entrepreneurial exposure and entrepreneurial action, moderated by entrepreneurial competencies (ECs). This paper draws from two psychology theories to develop and test a three-factor model of entrepreneurial action. The structuration theory formulates a theoretical model that explains how entrepreneurs’ interaction with their environment, and their concomitantly learned behavioral scripts (i.e., entrepreneurial competencies), impacts a newly formulated typology of entrepreneurial gestation activities based on the mindset theory of action phases. Furthermore, the ECs in this paper are drawn from a systematic framework of entrepreneurship competency development, which categorizes ECs into (1) entrepreneurial attitudes and personal characteristics and (2) entrepreneurial motives. By dividing entrepreneurial action into a predecisional, preactional, and actional phase, a novel approach is used in taking the context of the entrepreneurial process into account. It is proposed that prior entrepreneurial exposure is a significant and positive predictor of future entrepreneurial action in the predecisional and preactional phases. However, once entering the actional phase, this factor is no longer important, as women entrepreneurs have crossed the entrepreneurial Rubicon. The sample consists of South African entrepreneurs of which 346 women entrepreneurs and a sample of 804 male entrepreneurs are used to compare the results of the first hypothesis. Structural equation modeling (SEM) is used to model the relationship between prior entrepreneurial exposure and entrepreneurial action. Results confirm that prior entrepreneurial exposure in the form of role models, entrepreneurial parents, or any other form of exposure to entrepreneurship before starting a business is particularly important to encourage women to pursue business start-up (action). Furthermore, the development of certain ECs is crucial for improving the strength of the relationship between prior entrepreneurial exposure and entrepreneurial action for women entrepreneurs. These results have important implications for women entrepreneurs, educators, as well as entrepreneurship models, which have been traditionally male dominated.

## Introduction

Despite the acknowledged differences between men and women regarding their entrepreneurial action levels (Gupta et al., 2009), few have given specific scholarly attention to the amelioration of entrepreneurial action among women, with most research and models in entrepreneurship devoting more attention to their male counterparts (Wiklund et al., 2017). Globally, men have been reported to be twice as likely to start a business relative to women (Acs et al., 2005). While in developing country contexts, such as South Africa, this ratio improves, there still remains a significant discrepancy (Singer et al., 2018). Therefore, this requires research attention (Herrington et al., 2017), particularly given the important role that women entrepreneurs play in economic growth and poverty reduction (Kelley et al., 2015).

In addition to the lack of models specifically focusing on women entrepreneurs, scholars have also typically investigated antecedents of business start-up, such as entrepreneurial intentions (EI) and self-efficacy, constructs that only predict business start-up (entrepreneurial action) for a small minority of individuals (Kautonen et al., 2015; Shepherd et al., 2019). This is specifically true for women entrepreneurs, with research showing that women are not only less likely to form EI but are also significantly less likely to act on their EI (Shinnar et al., 2018). To this end, there remains a critical need to investigate the “complex nature of the female entrepreneurial endeavor” (Henry et al., 2016, 236), to explain and promote actual start-up behavior (Shinnar et al., 2018). Furthermore, to draw useful insights, it is important to consider women entrepreneurs’ interaction with the environment as a contextual factor that may explain their entrepreneurial endeavors, which few scholars have done from an objectivist epistemological stance to date (Henry et al., 2016).

Therefore, there is an urgent need to understand which factors contribute to the actual business start-up behavior (better known as entrepreneurial action) of women entrepreneurs, particularly from a developing country perspective. One of these factors could be prior entrepreneurial exposure. Previous scholars such as Kassean et al. (2015) found that aspiring entrepreneurs are more likely to start businesses when they learn from existing entrepreneurs through prior entrepreneurial exposure in the form of role models, “shadowing” the entrepreneur or having entrepreneurial family members. Therefore, this paper investigates the relationship between prior entrepreneurial exposure and entrepreneurial action. Given the importance of incorporating context and emphasizing models explaining the women entrepreneurial endeavor (Henry et al., 2016), the focus is on a sample of 346 women entrepreneurs in South Africa. By dividing the entrepreneurial action activities into a predecisional, preactional, and actional phase, a novel approach is used in taking the context of the entrepreneurial process into account (Delanoë-Gueguen and Fayolle, 2018).

Previous scholars such as McClelland (1973) as well as Wesselink and Wals (2011) suggest that entrepreneurship competency development should be done by means of a systematic approach. Jain (2011) followed such a systematic approach and conducted a meta-analysis on entrepreneurial competency research whereby entrepreneurial attitudes and personal characteristics as well as entrepreneurial motives make up entrepreneurial competencies (ECs). Following Jain’s (2011) framework, it shows how entrepreneurial intention and behavior affects or leads to entrepreneurial performance and outcomes. Therefore, we build on this work by focusing on specific ECs in this framework: for example, the need for achievement (motivation) is categorized as entrepreneurial motives (McClelland, 1961; Jain, 2011), and leadership, curiosity, self-efficacy, and innovativeness are categorized as entrepreneurial attitudes and personal characteristics (Jain, 2011; Strobl et al., 2012).

At the same time Morris et al. (2013) emphasize that there is a positive relationship between ECs and action. The theoretical model in this paper was found to fit the data, enabling the exploration of the moderating effects of certain ECs. As indicated above, these ECs include entrepreneurial attitudes and personal characteristics in the form of self-efficacy, leadership, curiosity, innovativeness, and entrepreneurial motives in the form of need for achievement (motivation). For all of the competencies, a moderating effect was observed, which confirms the following findings: (1) Prior entrepreneurial exposure in the form of role models, entrepreneurial parents, or any other form of exposure to entrepreneurship before starting a business is important when women pursue a business start-up, and (2) developing ECs from a systematic framework are crucial for improving the strength of the relationship between prior entrepreneurial exposure and entrepreneurial action. Structural equation modeling (SEM) was employed on a sample of women entrepreneurs and then compared to a male sample of 804 entrepreneurs in South Africa. Multigroup confirmatory factor analysis (CFA) was performed as an alternative method for assessing the effect of moderator variables in the model as an additional *post hoc* analysis. Interestingly, there was no model fit between prior entrepreneurial exposure and entrepreneurial action for the male sample. Hence, consistent with the notion that theoretical and empirical work needs distinct models explaining the female entrepreneurial endeavor (Bullough et al., 2017; Dy et al., 2017; Yang and del Carmen Triana, 2017), this paper demonstrates that a “one size fits all” approach cannot be applied to all entrepreneurs and that women entrepreneurs deserve unique scholarly attention. These findings confirm the results of other research that women entrepreneurs differ from male entrepreneurs in terms of business start-up (Wiklund et al., 2017). Thus, these findings contribute to the puzzle of why scholars observe lower EI and start-up behavior among women (Gupta et al., 2009; Shinnar et al., 2018) and how this may be ameliorated through ECs and prior entrepreneurial exposure.

Based on the results of this model, the following contributions to both the entrepreneurship and psychology literatures are advanced. First, from a theoretical standpoint, the impact of prior entrepreneurial exposure on the different phases of action and how various ECs may moderate these relationships are illustrated. As a result, the model validates the value of incorporating both psychology theories, namely the structuration and mindset theories for jointly and, respectively investigating ECs (Morris et al., 2013) and entrepreneurial action as a process (McMullen and Dimov, 2013). Second, this study sheds light on how the two categories of ECs can be used to ameliorate the entrepreneurial action levels of women entrepreneurs in developing countries. This is an important contribution given the relatively lower business start-up rates of women (Acs et al., 2005), as well as the poor start-up rates in developing country contexts (Herrington et al., 2017). Finally, from a practical standpoint, by providing an understanding of how prior entrepreneurial exposure and ECs interact to impact the different action phases of the entrepreneurial process, this research is of value to the various initiatives seeking to enable women entrepreneurs through various experiential activities and EC developmental training programs.

## Theoretical Foundation and Hypotheses Development

### Women and Men Entrepreneurs in South Africa

As South Africa is chronically faced with high levels of unemployment and underemployment, the persistent trend of low entrepreneurial action levels is of serious concern. Entrepreneurial action in South Africa has dropped by 25% in 2016 relative to 2013 (Herrington et al., 2017). Adding to the concern is the fact that entrepreneurial activity in South Africa is substantially below its African counterparts as well as other efficiency-driven economies (Herrington et al., 2017). It is acknowledged that due to contextual and support differences (Kew et al., 2013), a distinction should be drawn between developing countries such as South Africa and the developed countries such as Germany in terms of their entrepreneurial action levels (Singer et al., 2018).

Nevertheless, the current state of entrepreneurial activity is dire, and an important topic for scholars is how to enhance entrepreneurial action (business start-ups), particularly in South Africa (Herrington and Kelley, 2012; Kew et al., 2013; Herrington et al., 2017). In this regard, despite scholars acknowledging the differences between men and women regarding their entrepreneurial action levels (Gupta et al., 2009), few have given specific scholarly attention to the amelioration of entrepreneurial action among women, with most research and models in entrepreneurship devoting more attention to their male counterparts (Wiklund et al., 2017). Globally, men have been reported to be twice as likely to start a business relative to women (Acs et al., 2005). While in developing country contexts, such as South Africa, this ratio improves, there still remains a substantial discrepancy (Singer et al., 2018), which requires research attention (Herrington et al., 2017). This is imperative, given the important role that this group plays in economic growth and poverty reduction (Kelley et al., 2015), as well as the South African Government’s goal of women empowerment through self-employment (Herrington et al., 2017). As indicated previously, prior entrepreneurial exposure could enhance this self-employment (entrepreneurial action) for women entrepreneurs.

### The Relationship Between Prior Entrepreneurial Exposure and Entrepreneurial Action for Women Entrepreneurs

An emerging conviction among scholars is that there remains a critical need to investigate the “complex nature of the female entrepreneurial endeavor” (Henry et al., 2016, 236), to explain and promote their actual start-up behavior (Shinnar et al., 2018). Previous scholars have found that women are not only less likely to form EI but are also significantly less likely to act on their intentions (Shinnar et al., 2018). The above highlights the limitations of current models seeking to explain and ameliorate entrepreneurial action, particularly for women (Kautonen et al., 2015; Shepherd et al., 2019). According to the structuration theory (Giddens, 1984), entrepreneurs’ interaction with their environment, and their concomitantly learned behavioral scripts (i.e., entrepreneurial competencies), will impact entrepreneurial behavior.

#### Gender Differences in the Relationship Between Prior Entrepreneurial Exposure and Action

To this end, women entrepreneurs’ prior interaction with the environment may serve as an important contextual factor that may explain their entrepreneurial endeavors. While this notion has been explored from a constructionist epistemological stance, to date, few scholars have explored such a notion from a critical realist (objectivist) epistemological stance (Henry et al., 2016). In particular, prior entrepreneurial exposure has been cited as an important contextual factor that enhances aspiring entrepreneurs’ likelihood of starting businesses due to the learning that it provides, which forms guidelines of how to behave entrepreneurially (Mitchelmore and Rowley, 2010; Kassean et al., 2015).

Prior entrepreneurial exposure can come from existing entrepreneurs in the form of role models, “shadowing” the entrepreneur, having entrepreneurial family members, or prior work experience in an entrepreneurial firm (Hsu et al., 2017). Previous research has generally found a positive association between prior exposure and future entrepreneurial behavior (Schroder and Schmitt-Rodermund, 2006; Gielnik et al., 2018). This is consistent with the predictions of structuration theory (Giddens, 1984) that, by observing others, a prospective entrepreneur is able to develop behavioral scripts, which serve as a guide for future actions (Manz and Sims, 1980).

In particular, it is argued that prior entrepreneurial exposure is a more important antecedent for entrepreneurial action among women than among men. Women typically face significant challenges in terms of conflicting identity roles between traditional women roles and the traditionally male-dominated roles of entrepreneurship (Aggestam and Wigren-Kristoferson, 2017). These tensions often inhibit entrepreneurial behavior (Wang, 2019), as it forms another obstacle hindering feasibility perceptions of entrepreneurship as a career prospect (McMullen and Shepherd, 2006). Prior entrepreneurial exposure in the form of role models, entrepreneurial parents, or any other form of exposure to an entrepreneur before starting a business is therefore likely to significantly encourage women to pursue a business start-up (Kassean et al., 2015). Even more so, it is expected that women will respond more strongly if the role model or entrepreneur is female. Robb et al. (2014) found that women are more encouraged to start businesses if they were exposed to successful female entrepreneurs who can share stories and insights from their successes (and challenges).

Based on structuration theory (Giddens, 1984), this exposure will develop improved behavioral scripts for dealing with the contextual and role demands as a women entrepreneur and thereby enhance entrepreneurial behavior. Based on the above, the following hypothesis is put forward:

H1: There are stronger statistically significant paths for the women entrepreneur sample than the men entrepreneur sample in the relationship between prior entrepreneurial exposure and action.

#### The Rubicon Crossing According to the Mindset Theory of Action Phases

In addition to the above, the mindset theory of action phases also proposes that, as individuals progress through the various phases toward their goal, their mindset evolves (Gollwitzer, 2012; Delanoë-Gueguen and Fayolle, 2018). Therefore, it is important to breakdown entrepreneurial action into its constituent phases to more clearly understand the impact of prior entrepreneurial exposure on action. According to this theory, entrepreneurial action can be divided into a predecisional, preactional, and actional phase (Delanoë-Gueguen and Fayolle, 2018).

As stated previously, current intention-based entrepreneurship models argue that the stronger the EI, the higher the action likelihood should be. This view aligns with the motivational (predecisional) phase of the Rubicon model of action phases (Delanoë-Gueguen and Fayolle, 2018). Following this phase, to achieve concrete action, a distinct switch from a goal (i.e., predecisional and preactional phase) to an implementation intention (i.e., actional phase) is required (Brandstätter et al., 2003; Gollwitzer, 2012). According to Brandstätter et al. (2003), for non-routine goals such as entrepreneurial pursuits, a series of goal-directed actions must be initiated to achieve the goal. Particularly for entrepreneurial endeavors, progress through this process can be identified by the types of gestation activities undertaken by the individual (Teague and Gartner, 2017).

From the standpoint of the Rubicon model, these types of activities can be identified and used to determine when a potential entrepreneur crosses the entrepreneurial Rubicon (i.e., engaged in predecisional and preactional phases) to become a nascent entrepreneur who is no longer acting from motivation, but rather volition (Heckhausen, 2000). In fact, a recent work by Delanoë-Gueguen and Fayolle (2018) conclusively demonstrates the existence of this Rubicon for entrepreneurs. A major implication of this model is that once individuals cross the Rubicon, the ability of intentions to explain action should disappear (Delanoë-Gueguen and Fayolle, 2018).

Consequently, based on this theory, once women entrepreneurs cross the entrepreneurial Rubicon and enter into the actional phase, motivational factors will no longer matter as the action is driven by volition (Heckhausen, 2000). Hence, it is proposed that prior entrepreneurial exposure is a significant and positive predictor of future entrepreneurial action in the predecisional and preactional phases. However, once entering the actional phase, this factor is no longer important, as they have crossed the entrepreneurial Rubicon. From the above, and with the objective of generating a better understanding of how to positively encourage women entrepreneurship, the following hypotheses are put forward:

H2: Prior entrepreneurial exposure will have statistically significant paths for the predecisional and preactional phases of entrepreneurial action for women entrepreneurs.
H3: Prior entrepreneurial exposure will not have a statistically significant path for the actional phase of entrepreneurial action for women entrepreneurs.

### The Moderating Role of Entrepreneurial Competencies

Entrepreneurial competencies refer to the knowledge, skills, and abilities that contribute toward entrepreneurial action and improved performance (Man et al., 2008). Consequently, scholars have shown that psychological and cognitive characteristics such as attitudes, motives, self-efficacy, and other personal factors influence entrepreneurial action (Morris et al., 2013; Santos et al., 2013). Jain (2011) followed a systematic approach by developing an entrepreneurial competency framework. In this framework, ECs are categorized as (1) entrepreneurial attitudes and personal characteristics and (2) entrepreneurial motives. We build on this work by drawing individual ECs from the two categories from this framework. Specifically, the need for achievement (motivation) is categorized as an entrepreneurial motive competency (McClelland, 1961). Curiosity, leadership, self-efficacy, and innovativeness are categorized as entrepreneurial attitudes and personal characteristics competencies (Strobl et al., 2012). Therefore, these five individual ECs were developed and tested in this paper. At the same time, Morris et al. (2013) emphasize that there is a positive relationship between ECs and action. In particular, these ECs may play an important moderating role for women entrepreneurs in the relationship between entrepreneurial exposure and action.

In addition, based on the predictions of the Rubicon Model (Delanoë-Gueguen and Fayolle, 2018), the ECs likely have a moderating effect on the motivational phases of predecision and preaction but not on the volitional phase of action. This is because once the Rubicon has been crossed into action, motivational predictors are unlikely to influence action (Brandstätter et al., 2003; Gollwitzer, 2012). This leads to the hypotheses statements in the sections that follow.

#### ECs Categorized as Entrepreneurial Attitudes and Personal Characteristics

##### Leadership as a Moderating Variable

Leadership refers to the action of leading a group of people or an organization (Yıldız et al., 2014). It entails developing a vision, sharing the vision, and encourage others to follow the stated vision (Morris et al., 2013). Entrepreneurship is a special case of leadership and is distinguished from other forms of leadership in terms of the need to convey an entirely new vision for an emerging venture rather than an existing vision for an existing business (Morris et al., 2013). Wells (2014) states that an effective entrepreneurial leader is a person who practices lifelong learning, self-direction, and builds nurturing relationships with others to achieve a common goal. Consequently, the ability to effectively lead has been suggested as an essential element of taking productive entrepreneurial action (Santos et al., 2013). An individual with strong leadership competencies is likely to be able to more readily transfer the benefits of prior entrepreneurial exposure to entrepreneurial action, with empirical evidence supporting the importance of this EC to action (Kyndt and Baert, 2015). Wells (2014) states that this is even more so for women entrepreneurs who need to acquire and enhance their leadership competencies in order to direct their ventures and successfully meet the challenges of complex and rapidly changing entrepreneurial business environments. It is, thus, hypothesized that:

H4: Leadership has a moderating effect on the relationship such that the positive relationship between prior entrepreneurial exposure and the predecisional and preactional phases of entrepreneurial action will be stronger when women entrepreneurs possess more leadership.

##### Innovativeness as a Moderating Variable

Innovativeness has been cited as an important EC that may enhance entrepreneurial action levels (Morris et al., 2013; Santos et al., 2013). According to Morris et al. (2013) innovativeness refers to the “capabilities of developing new products, services, and/or business models that generate revenues exceeding their costs and produce sufficient user benefits to bring about a fair return.” Scholars such as Gorman et al. (1997); Feldman and Bolino (2000), and Sternberg (2004) have long suggested that innovative individuals are motivated to become self-employed. For example, Groves et al. (2008) found that individuals who had taken significant entrepreneurial action exhibited higher levels of non-linear thinking and innovativeness. This has led scholars such as Cha and Bae (2010) to propose that the predictive value of innovativeness lies in it enhancing individuals’ intention to adopt innovative activities, such as taking entrepreneurial action. Coleman and Robb (2012) is of the opinion that women who have experience in prior entrepreneurial exposure, “developed innovative firms as they ‘know the ropes,’ and they can provide guidance and encouragement to women who are just getting started.” This leads to the following hypothesis:

H5: Innovativeness has a moderating effect on the relationship such that the positive relationship between prior entrepreneurial exposure and the predecisional and preactional phases of entrepreneurial action will be stronger when women entrepreneurs possess more innovativeness.

##### Curiosity as a Moderating Variable

Curiosity refers to the catalyst ingredient, which leads entrepreneurs to pry into *status quo* products and services to find new solutions to better solve customers’ problems (Steyaert et al., 2011). A sense of curiosity means you look at even the smallest problems and seek a better solution (Fletcher, 2011). Curiosity can further be described as finding answers, which enables a person to improve decision making. An entrepreneur that has a high level of curiosity will be interested in understanding how the economy works, how to improve results, and how business works (Fletcher, 2011). Therefore, there is likely a strong link between entrepreneurial curiosity and entrepreneurial action, particularly since it will encourage the individual to (a) explore new options that could create additional profit, (b) develop interest for other entrepreneurial opportunities, (c) research new opportunities, and (d) develop new solutions for current problems in business (Fletcher, 2011). Thus, a curious individual with prior entrepreneurial exposure is more likely to engage in entrepreneurial action because their interest likely allows them to identify and exploit opportunities based off of this experience more readily (McMullen and Shepherd, 2006). Moreover, a study conducted by Strobl et al. (2012) found that innovativeness, knowledge, and curiosity and desire for independence affect women EI in a positive way. This leads to the following hypothesis:

H6: Curiosity has a moderating effect on the relationship such that the positive relationship between prior entrepreneurial exposure and the predecisional and preactional phases of entrepreneurial action will be stronger when women entrepreneurs possess more curiosity.

##### Self-Efficacy as a Moderating Variable

Self-efficacy refers to the perceived capability to perform certain tasks (Kassean et al., 2015). Self-efficacy is a motivational construct that has been shown to influence an individual’s choice of activities, goal levels, persistence, and performance in a range of contexts (Zhao et al., 2005). Individuals with a high level of self-efficacy tend to set challenging goals, persist even in the face of failure and approach difficult tasks as challenges to be mastered rather than issues to be avoided (Botha and Bignotti, 2017). In this regard, the construct has been shown to be an important driver of entrepreneurial action (Mitchell and Shepherd, 2010; Kyndt and Baert, 2015), particularly as a moderator of the relationship between EI and entrepreneurial action (Boyd and Vozikis, 1994). Self-efficacy can be strengthened through prior experience, having role models, receiving words of encouragement, and positive well-being (Sweida, 2018). Therefore, for entrepreneurial experience to have significantly positive impact of entrepreneurial action, it is likely that an individual needs to perceive themselves as having sufficient self-efficacy (Schlaegel and Koenig, 2014). In the study by Sweida (2018), the Social Cognitive Theory has supported the finding that self-efficacy had a significant positive effect on women entrepreneurs’ business performance. This leads to the following hypothesis:

H7: Self-efficacy has a moderating effect on the relationship such that the positive relationship between prior entrepreneurial exposure and the predecisional and preactional phases of entrepreneurial action will be stronger when women entrepreneurs possess more self-efficacy.

#### EC Categorized as Entrepreneurial Motives

##### Motivation (Need for Achievement) as a Moderating Variable

Need for achievement refers to an individual’s desire for significant accomplishment, mastering of skills, control, or high standards (Jain, 2011; Chen et al., 2012). Since as early as McClelland (1961), scholars have asserted that entrepreneurs have a distinctly higher need for achievement than others (Zeffane, 2013). Need for achievement emerges as an important unmet need that requires satisfaction, often manifesting through entrepreneurial behavior (Lam et al., 2017). Consequently, since an individual’s existing frame of reference is put against the individual’s own desire to achieve (McClelland, 1961), it is likely that an individual will consider their current level of prior entrepreneurial exposure and, in light of the motivation, will engage in varying levels of entrepreneurial action. In particular, higher motivation should result in greater action. Furthermore, Ramadani et al. (2018) suggest that the motivation of women entrepreneurs is freedom in decision-making, profit, need for achievement, and the opportunity to work exclusively for themselves as the main motives linked to entrepreneurial action. In this sense, the following hypothesis is put forward:

H8: Motivation has a moderating effect on the relationship such that the positive relationship between prior entrepreneurial exposure and the predecisional and preactional phases of entrepreneurial action will be stronger when women entrepreneurs possess more motivation.

**FIGURE 1:**
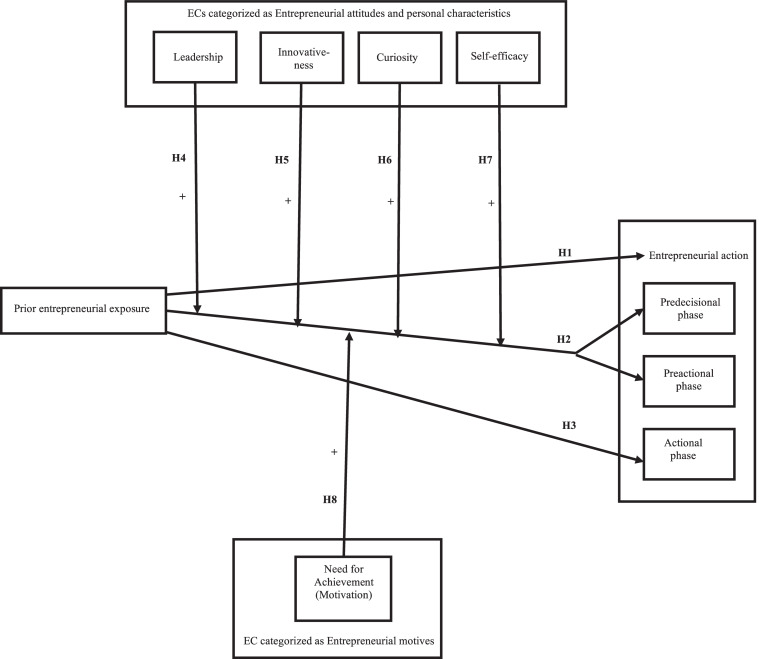
Hypothesized model for women entrepreneurs.

Given the above discussion, the hypothesized relationships are depicted in Figure 1 below.

## Materials and Methods

### Sample and Procedure

The target population for this study consisted of existing women entrepreneurs who had commenced business operations and were still operating a business venture in 2019 in South Africa. The units of analysis and observation were the individual existing women entrepreneurs. Since a male entrepreneur sample was also added to compare the results of the first hypothesis, the total target population for this study was existing entrepreneurs in South Africa, and a sampling frame could not be feasibly obtained. Consequently, a non-probability sampling method, specifically proportionate quota sampling, was used (Saunders et al., 2016).

In order to ensure representativeness, the industry in which the entrepreneur’s main business operates (i.e., their industry background) and the age of the entrepreneurs’ businesses (start-up or established) were used as quota control variables for this research study. The online survey link was emailed to a database of 20,000 entrepreneurs from a range of industries and all nine provinces in South Africa. Data for the main study were collected over a 3-month period from December 2018 to February 2019. In total, the final sample consists of 1,150 usable surveys (5.75% response rate) that were collected, with 346 female and 804 male respondents. As indicated in the theoretical foundation, gender appears to be related to entrepreneurial activity in South Africa and may potentially play a role in entrepreneurial action among existing entrepreneurs (Herrington et al., 2017). For the purpose of this paper, the women sample’s findings will be explained in the “Results” section as the paper focuses on women entrepreneurs. Additionally, the male sample will only be used to compare the results of the first hypothesis, which states that: There are stronger statistically significant paths for the women entrepreneur sample than the men entrepreneur sample in the relationship between prior entrepreneurial exposure and action. Based on the result of this hypothesis, the women entrepreneur sample is considered independently for the remaining hypotheses.

### Measures

#### Independent Variable

Prior entrepreneurial exposure (such as whether the respondents had worked in an entrepreneurial business before, have family members that owns businesses, or have entrepreneurial role models) consists of four items measured on a 5-point Likert-type scale where respondents were asked to rate their level of agreement with each of the statements, ranging from 1 (strongly disagree) to 5 (strongly agree). This scale was adopted from the scale by Peterman and Kennedy (2003).

#### Moderator Variables

Independent scales were used to measure each of the five ECs. All of the entrepreneurial competency scales, except the self-efficacy scale, consist of four/five items to measure each of the competencies on a 7-point Likert-type scale where respondents were asked to rate their level of agreement with each statement, ranging from 1 (strongly disagree) to 7 (strongly agree).

The innovativeness competency comprises of four items from the scale by Lukas and Ferrell (2000) as well as by Mitchelmore and Rowley (2010). The need for achievement (motivation) competency comprises of five items from the scale by Lynn (1969). The curiosity competency comprises of four items from the scale by Fletcher (2011). The leadership competency comprises of four items from the scale by Rush et al. (1977). The self-efficacy competency comprises of five items from the scale by Siu et al. (2005) and was measured on a 4-point Likert-type scale where respondents were asked to rate their level of agreement with each statement, ranging from 1 (not at all true) to 4 (exactly true). All the scales were adapted and simplified to fit the targeted South African population.

#### Dependent Variable

The entrepreneurial action scale (McMullen and Shepherd, 2006) consists of 17 items to measure the business start-up activities on a 5-point Likert-type scale where respondents were asked to what extent they have engaged in the listed activities toward venture creation in the last 3 years, ranging from 1 (never) to 5 (always). Factor analysis grouped the activities into the three actional phases outlined by the entrepreneurial Rubicon Model (Delanoë-Gueguen and Fayolle, 2018).

### Validity and Reliability

Confirmatory factor analysis was used to confirm construct validity for all the EC scales. The Cronbach alpha coefficient was used to determine internal consistency (reliability). SEM was used to model the relationship between prior entrepreneurial exposure and action using AMOS V25. It allows to test research hypotheses in a single process by modeling complex relationships among many observed and latent variables. Goodness of fit will be assessed through an acknowledged set of fit indices and associated thresholds. The first index is the chi-square ratio (*x*^2^/*df*): A value of <3 is seen as an acceptable fit, while values <5 indicate a reasonable fit. For the Incremental Fit index (IFI) and the Comparative Fit index (CFI), a value of 0 reflects no fit, while a value of 1 reflects a perfect fit. Values above or equal to 0.90 reflect an acceptable fit. For roots mean squared error of approximation (RMSEA), a value of 0.05 represents a close approximate fit, while values between 0.05 and 0.08 suggest a reasonably approximate fit, and values >0.10 suggest a poor fit.

### Assessment of Moderation

The multigroup CFA has been used as the method for assessing the effect of moderating variables in the model. The path of interest where the moderator variable is to be assessed is constrained with parameter = 1, and the model is termed as the constrained model. The procedure will estimate two models separately. One is the *constrained model*, while the other one is the *unconstrained model*. If the difference between the chi-square values of the constrained and unconstrained model is more than 3.84, then moderation has occurred in the model (Awang, 2012).

## Results

### Demographic and Business Profile of Women Entrepreneurs

The total sample comprises of 1,150 respondents. Most of the respondents are male (69.9%), have a postgraduate degree (39.9%), and are between the ages of 54 and 65 (34.9%). Regarding their businesses, most of the respondents operate a service-based business (48.3%), have an annual turnover of 0 to R1,000,000 (32.7%); break-even occurred longer than 1 year ago (47.3%) and operates their businesses in the Gauteng province (49.3%) of South Africa.

Focusing on women, the sample comprises of 346 women entrepreneurs. The average age is 50 years with the youngest respondent being 26 years and the oldest being 78 years. Just over half (51.1%) of these respondents are in possession of at least an undergraduate degree (17.6% has an undergraduate degree and 33.5% a postgraduate degree). The majority of the businesses (56.6%) is service based, mainly in the Gauteng or Western Cape provinces (70.3%) and predominately in the financial, manufacturing, and business services industries (31.7%). On average, the businesses are 5 years old, and with regard to time to break even, the modal category is longer than 1 year (42.8%). Furthermore, approximately three quarters of the respondents (75.2%) businesses have a turnover of <5 million Rand. As the focus on this paper is on women entrepreneurs, CFA, the descriptive statistics, and Cronbach alpha values are presented in the next sections on the women entrepreneur sample only.

### Validity and Reliability of the Women Entrepreneur Sample

The results of the CFA fit indices are presented in Tables 1, 2 confirmed the unidimensionality of each of the EC constructs. Four items loaded <0.5 (two from the original four-item leadership scale, one from the original five-item self-efficacy scale, and one from the original five-item motivation scale) in the confirmatory analysis incorporating all items and were deleted in the final CFA. In Table 3, three factors were identified for entrepreneurial action and subsequently labeled as predecisional (EA1 – factor 1), preactional (EA2 – factor 2), and actional (EA3 – factor 3) phases. For prior entrepreneurial exposure, in Table 4, it is evident that one factor emerged.

**TABLE 1:** Confirmatory factor analysis fit indices.

Models	χ^2^/*df*	IFI	*CFI*	RMSEA
Model 1 (all original items)	3.012	0.859	0.857	0.076
Model 2 (4 items deleted)	2.818	0.903	0.902	0.073

**TABLE 2:** Summary of the confirmatory factor analysis for the individual EC constructs.

Construct	Item description	Factor
Leadership	I let employees know what is expected of them.	0.748
	I put suggestions made by the group into operation.	0.742
Innovativeness	I proactively create new opportunities and respond to change relative to new products.	0.765
	I continuously create new products or services and/or enhance old ones.	0.851
	I constantly develop products of services appropriate to the firms chosen market niche/product innovativeness.	0.792
	I have developed a number of new-to-the market ideas.	0.696
Curiosity	I explore new things that could create additional profit.	0.672
	I am interested in other entrepreneurial opportunities.	0.635
	When I have some free time, I spend it researching new opportunities.	0.726
	Problems related to entrepreneurship encourage me to look for solutions.	0.747
Self-efficacy	I can always manage to solve difficult problems if I try hard enough.	0.496
	It is easy for me to stick to my aims and accomplish my goals.	0.543
	I am confident that I could deal efficiently with unexpected events.	0.797
	I can remain calm when facing difficulties because I can rely on my coping abilities.	0.679
Motivation (need for	I have always worked hard in order to be the best in my own line of business.	0.670
achievement)	I always strive for excellence in everything that I do.	0.712
	The most important thing to me is succeeding in what I do.	0.729
	I set ambitious goals and work hard to achieve them.	0.768

**TABLE 3:** Summary of the factor analysis for the entrepreneurial action construct.

Construct	Item description	KMO and Bartlett Test	Variance explained	Factor 1	Factor 2	Factor 3
Entrepreneurial action	I have spent a lot of time thinking about starting a business before I actually started my business.	0.842 (*p* < 0.001)	59.7%	0.559		
	I have organized a start-up team.			0.732		
	I have identified market opportunities.			0.642		
	I have prepared a business plan.			0.543		
	I have selected a business name.				0.587	
	I have created a legal entity.				0.687	
	I have registered with the tax authorities.				0.723	
	I have invested some of my own money in a business.				0.385	
	I have requested for and received financial assistance to start my business.					0.392
	I have facilities and equipment in place that assisted me in starting a business.					0.704
	I have purchased or leased major items, like equipment, facilities or property.					0.674
	I have purchased raw materials, inventory, or other supply.					0.559

**TABLE 4:** Summary of the factor analysis for the prior entrepreneurial exposure construct.

Construct	Item description	KMO and Bartlett Test	Variance explained	Factor
Prior entrepreneurial	My parents currently own or have owned a business.	0.755 (*p* < 0.001)	56.2%	0.662
exposure	A family member/s other than my parents currently own or have owned a business.			0.706
	I have worked in a family business before.			0.680
	Other than my parents or other family members, I have an entrepreneurial role-model.			0.531

*The descriptive statistics, Cronbach alpha values, and correlation for all the constructs are presented in Table 5 below.*

All constructs had a Cronbach Alpha value above 0.7, the recommended thresholds for established constructs (Field, 2018). It is evident from Table 5 that all the ECs were weakly to moderately correlated except for a strong correlation between curiosity and innovativeness (0.603).

**TABLE 5:** Descriptive statistics, Cronbach alpha values, and correlations for the moderating, dependent and independent variables.

	M	SD	Alpha	Leadership	Innovativeness	Curiosity	Self-efficacy	Motivation	Predecisional (EA1)	Preactional (EA2)	Actional (EA3)	Prior entrepre neurial exposure
Leadership	5.87	0.87	0.713	*1*								
Innovativeness	5.41	1.04	0.849	0.438**	*1*							
Curiosity	5.87	0.86	0.787	0.395**	0.603**	*1*						
Self-efficacy	3.26	0.43	0.713	0.280**	0.316**	0.303**	*1*					
Motivation	6.31	0.65	0.797	0.475**	0.472**	0.462**	0.399**	*1*				
Predecisional (EA1)	3.35	0.99	0.740	0.336**	0.347**	0.366**	0.247**	0.234**	*1*			
Preactional (EA2)	3.78	0.85	0.716	0.361**	0.363**	0.284**	0.257**	0.230**	0.466**	*1*		
Actional (EA3)	4.40	0.76	0.719	0.287**	0.289**	0.280**	0.231**	0.246**	0.437**	0.458**	*1*	
Prior entrepreneurial exposure	2.73	1.25	0.739	0.009	0.041	0.110**	0.018	−0.018	0.153**	0.108*	−0.008	*1*

*Significance at the 5% level (*p* < 0.05). **Significance at the 1% level (*p* < 0.01).

### Structural Model Results

In Table 6, the model fit indices for the relationship between entrepreneurial exposure and entrepreneurial action were first assessed for the men and women samples, respectively, without moderation effects. This was done in order to establish the feasibility of moderation testing, which is conducted in Table 7. No alternative models were considered appropriate for each gender group.

**TABLE 6:** Model fit indices.

Models	χ^2^/*df*	IFI	*CFI*	RMSEA
Model 1 (men)	3.181	0.947	0.946	0.052
Model 2 (women)	2.330	0.924	0.922	0.062

**TABLE 7:** Multigroup moderation tests.

Moderator	Groups	Path	Constrained	Unconstrained	Chi-square difference
Leadership	Low leadership	Exp to EA1	176.6	155	21.6*
		Exp to EA2	181.6	155	26.6*
	High leadership	Exp to EA1	116	95.5	20.5*
		Exp to EA2	125.7	95.5	30.2*
Innovativeness	Low innovativeness	Exp to EA1	146.7	116.9	29.8*
		Exp to EA2	143.9	116.9	27*
	High innovativeness	Exp to EA1	136.4	127.2	9.2*
		Exp to EA2	151.8	127.2	24.6*
Curiosity	Low curiosity	Exp to EA1	175.7	148.4	27.3*
		Exp to EA2	182.5	148.4	34.1*
	High curiosity	Exp to EA1	112.9	99.8	13.1*
		Exp to EA2	121.3	99.8	21.5*
Self-efficacy	Low self-efficacy	Exp to EA1	145.5	131.3	14.2*
		Exp to EA2	151.3	131.3	20*
	High self-efficacy	Exp to EA1	134.1	102.8	31.3*
		Exp to EA2	140.3	102.8	37.5*
Motivation	Low motivation	Exp to EA1	152.8	131.6	21.2*
		Exp to EA2	161.5	131.6	29.9*
	High motivation	Exp to EA1	119.2	102.5	16.7*
		Exp to EA2	124.6	102.5	22.1*

*Moderation is indicated by a value above 3.84.

The model fit statistics indicated adequate fit of the models (models 1 and 2): chi-square/*df* < 3 for women and very close to three for men, still smaller than 5, IFI and CFI > 0.9, and RMSEA < 0.08. It is important to note that both models 1 (men) and 2 (women) indicated model fit, and the chi-square values (not normed) were not compared for the two models as the moderation effect of gender was not tested. However, the results further indicates that, for the women sample, statistically significant paths, at the 5 and 10% level, respectively, of prior entrepreneurial exposure with EA1 (standardized β = 0.156, *p* < 0.05) and EA2 (β = 0.124, *p* < 0.1), but not with EA3 (β = −0.068, *p* > 0.1).

For the male sample, no statistically significant paths were observed for prior entrepreneurial exposure with EA1 (standardized β = −0.003, *p* > 0.1), EA2 (β = 0.01, *p* > 0.1), and EA3 (β = 0.002, *p* > 0.1). The result may appear to be contradictory for the male sample, given that the model fit was adequate, but there are no statistically significant structural paths. However, the lack of significant paths is consistent with the predictions of H1. It is important to note that, as the endogenous variables are correlated (EA1, EA2, and EA3) and these endogenous variables can only share variance that definitely belongs to them, i.e., the residual (or error) variance, these were correlated in the model and contributed to the fit of the model. Therefore, the male sample was not used to test the moderating effect of the five ECs on the relationship between the prior entrepreneurial exposure and entrepreneurial action factors (EA1 and EA2).

### Moderation Results of the Women Entrepreneur Sample

Following the multigroup CFA approach to test moderation, the results are indicated in Table 7.

The results indicate that, for women entrepreneurs, all five ECs are statistically significant moderators in the relationship between prior entrepreneurial exposure with entrepreneurial action (EA1 and EA2).

In Supplementary Addendum 1, moderation graphs for the women entrepreneur sample are illustrated. To interpret the moderator effect, prior entrepreneurial exposure was split at the median to form low and high subgroups. Then, each subgroup was plotted against average EA1 and EA2, respectively. Figures 1–10 (Supplementary Addendum 1) show the interaction effect for all the moderating variables. Therefore, interactive line graphs were used to explain the moderator effects.

In summary, in the case of EA1, all the ECs strengthen the relationship with prior entrepreneurial exposure except for self-efficacy, which appear to strengthen the relationship at low levels but do not have an impact at high levels of self-efficacy. In the case of EA2, the motivation and leadership competencies strengthen the relationship with prior entrepreneurial exposure, while it appears that for curiosity, self-efficacy, and innovation, the relationship weakens.

## Conclusion

As indicated previously, the male entrepreneur sample was included to test the first hypothesis (H1). This was conducted to determine whether women entrepreneurs have a stronger relationship between prior entrepreneurial exposure and action than their counterparts. The SEM and model fit indices indicated adequate model fit for both the men (model 1) as well as the women entrepreneur samples (model 2), respectively. However, the results further reveal that, for the women entrepreneur sample, statistically significant paths were observed for prior entrepreneurial exposure with the predecisional (EA1) and preactional (EA2) phases. This finding is supported by Aggestam and Wigren-Kristoferson (2017), Wang (2019), and McMullen and Shepherd (2006) in the literature. For the male sample, although the data fit the model, no statistically significant paths were observed for prior entrepreneurial exposure with EA1, EA2, and EA3. Therefore, H1 is accepted, as the lack of significant paths for the male sample is consistent with the predictions of H1. This finding indicates that prior entrepreneurial exposure in the form of role models or entrepreneurs encourage women entrepreneurs to a greater extent, than male entrepreneurs, to start businesses.

As the results indicate that statistically significant paths were observed for prior entrepreneurial exposure with the predecisional (EA1) and preactional (EA2) phases but not with the actional (EA3) phase for the women entrepreneur sample, H2 is accepted. This finding supports the notion of the Rubicon Crossing according to the Mindset Theory of Action Phases (Delanoë-Gueguen and Fayolle, 2018), which proposes that prior entrepreneurial exposure is a significant and positive predictor of future entrepreneurial action in the predecisional and preactional phases. At the same time, as statistically significant paths were not observed for prior entrepreneurial exposure with the actional (EA3) phase, there is support that once women entrepreneurs cross the entrepreneurial Rubicon and enter into the actional phase (EA3), motivational factors will no longer matter as the action is driven by desire or volition (Heckhausen, 2000). Therefore, H3 is also accepted, and this paper confirms that motivational factors such as prior entrepreneurial exposure is only significant for the phases that lead up to actual action, and thereafter, women entrepreneurs are driven by desirability. In order to enhance business start-up of women entrepreneurs, both motivational and desirability factors should be developed.

Based on the above, in order to test H4–H8, the multigroup CFA approach to test moderation was employed for prior entrepreneur exposure with EA1 and EA2 and not tested for EA3 as the path was not statistically significant. The findings reveal that all five the ECs (leadership – H4; innovativeness – H5; curiosity – H6; self-efficacy – H7; and motivation – H8) are statistically significant moderators in the relationship between prior entrepreneurial exposure with entrepreneurial action (EA1 and EA2) for women entrepreneurs.

When investigating the moderation graphs, it is evident that the positive relationship between prior entrepreneurial exposure and the predecisional and preactional phases of entrepreneurial action is stronger when women entrepreneurs possess more leadership (Santos et al., 2013; Wells, 2014; Kyndt and Baert, 2015) and need for achievement (motivation) (McClelland, 1961; Ramadani et al., 2018). More specifically, for the predecisional phase, innovativeness (Cha and Bae, 2010; Coleman and Robb, 2012; Robb et al., 2014) and curiosity (McMullen and Shepherd, 2006) also strengthened the relationship, whereas self-efficacy (Boyd and Vozikis, 1994; Schlaegel and Koenig, 2014; Sweida, 2018) only strengthened the relationship at low levels of self-efficacy. For the preactional phase, only leadership and motivation strengthened the relationship, whereas the other ECs did not.

## Discussion

Women entrepreneurs play an enormous role in economic growth and poverty reduction (Kelley et al., 2015). Yet, previous scholars such as Shinnar et al. (2018) found that women are not only less likely to form EI but are also significantly less likely to act on their EI. At the same time, there is a lack of models specifically focusing on women entrepreneurs, and most of these models only predict business start-up (entrepreneurial action) for a small minority of individuals (Kautonen et al., 2015; Shepherd et al., 2019). Scholars such as Henry et al. (2016) call for models to explain and promote actual start-up behavior for women entrepreneurs (Shinnar et al., 2018). In this paper, it is done from an objectivist epistemological stance, as there is an urgent need to understand which factors contribute to the actual business start-up behavior (better known as entrepreneurial action) of women entrepreneurs, particularly from a developing country perspective. Kassean et al. (2015) suggest that one of these factors could be prior entrepreneurial exposure where aspiring entrepreneurs are more likely to start businesses when they learn from existing entrepreneurs through prior entrepreneurial exposure in the form of role models or having entrepreneurial parents.

Therefore, this paper models the relationship between prior entrepreneurial exposure and entrepreneurial action on 346 women entrepreneurs in South Africa. This paper draws from two psychology theories, the structuration theory (Giddens, 1984) and the mindset theory of action phases (Gollwitzer, 2012; Delanoë-Gueguen and Fayolle, 2018) to develop and test a three-factor model of entrepreneurial action. By dividing the entrepreneurial action activities into a predecisional, preactional, and actional phase, a novel approach is used in taking the context of the entrepreneurial process into account (Delanoë-Gueguen and Fayolle, 2018).

Previous scholars such as Morris et al. (2013) emphasize that there is a positive relationship between ECs and action. Therefore, we build on Jain’s (2011) work by focusing on specific ECs in a systematic framework. In this framework, leadership, curiosity, self-efficacy, and innovativeness are categorized as entrepreneurial attitudes and personal characteristics (Jain, 2011; Strobl et al., 2012), and the need for achievement (motivation) is categorized as entrepreneurial motives (McClelland, 1961; Jain, 2011). SEM and multigroup CFA were employed on a sample of women entrepreneurs and then compared to a male sample of 804 entrepreneurs in South Africa. Interestingly, although there was a model fit between prior entrepreneurial exposure and entrepreneurial action for the male sample, there were no statistically significant paths. However, for the women entrepreneur sample, the theoretical model was found to fit the data and the exploration of the moderating effects of the following ECs, e.g., self-efficacy, leadership, curiosity, innovativeness, and need for achievement (motivation), were determined.

All the hypotheses stated in this paper are accepted. First, there are stronger statistically significant paths for the women entrepreneur sample than the men entrepreneur sample in the relationship between prior entrepreneurial exposure and action. This finding confirms that women entrepreneurs deserve unique scholarly attention and that women entrepreneurs differ from male entrepreneurs in terms of business start-up (Wiklund et al., 2017).

Furthermore, prior entrepreneurial exposure has statistically significant paths for the predecisional and preactional phases of entrepreneurial action for women entrepreneurs but not for the actional phase. This finding confirms and supports the Rubicon Crossing according to the Mindset Theory of Action Phases (Delanoë-Gueguen and Fayolle, 2018). Thus, these findings contribute to understanding why lower EI and start-up behavior is observed among women entrepreneurs (Gupta et al., 2009; Shinnar et al., 2018). However, this paper sheds light on the importance of developing both the entrepreneurial attitudes and personal characteristics as well as the entrepreneurial motives ECs for women entrepreneurs. Particularly, most of the five ECs, except self-efficacy, are crucial in strengthening the relationship between prior entrepreneurial exposure and entrepreneurial action. As self-efficacy is closely related to EI, this finding supports the work of previous scholars, which indicated that lower EI is observed among women entrepreneurs.

### Theoretical Contributions

This paper addresses the gap of exploring the antecedents in the form of prior entrepreneurial exposure and various ECs of entrepreneurial behavior (action and start-up) for women entrepreneurs. First, while entrepreneurial behavior has been explored from a constructionist epistemological stance, to date, few scholars have explored such a notion from a critical realist (objectivist) epistemological stance. From a theoretical view, the impact of prior entrepreneurial exposure on the different phases of entrepreneurial action and how various ECs may moderate these relationships are illustrated. This is an underresearched area, particularly for women entrepreneurs. As there is a lack of literature on the entrepreneurial action and prior entrepreneurial exposure of women entrepreneurs in developing countries, specifically South Africa, this paper contributes to the literature on this topic in a developing country context.

Second, as a result, the hypothesized model validates the value of incorporating both psychology theories, namely, the structuration and mindset theories for jointly and, respectively investigating ECs (Morris et al., 2013) and entrepreneurial action (McMullen and Dimov, 2013). As three factors emerged in this paper for entrepreneurial action, namely, predecisional, preactional, and actional phases, action is investigated from a process perspective. This finding might encourage other studies to focus on entrepreneurial action as a process rather than a stagnant construct.

Third, this study sheds light on how ECs can be used to ameliorate the entrepreneurial action phases of women entrepreneurs in developing countries. This is an important contribution given the relatively lower business start-up rates of women (Acs et al., 2005), as well as the poor start-up rates in developing country contexts (Herrington et al., 2017).

Finally, this paper contributes to the Rubicon Crossing literature as well as the Mindset Theory of Action Phases, as this paper confirms that prior entrepreneurial exposure is a significant and positive predictor of future entrepreneurial action phases, specifically the predecisional and preactional phases. However, once entering the actional phase, the women entrepreneurs in this sample have crossed the entrepreneurial Rubicon, and it is suggested that they are motivated by desire rather than other factors. From the above, and with the objective of generating a better understanding of how to positively encourage women entrepreneurship, this paper suggests that entrepreneurial action should be investigated from a process perspective in the form of three phases.

### Practical Implications

From a practical standpoint, by providing an understanding of how entrepreneurial action activities should be divided into a predecisional, preactional, and actional phase, for women entrepreneurs, a novel approach is used in taking the context of the entrepreneurial process into account (Delanoë-Gueguen and Fayolle, 2018). Educators and entrepreneurial development programs could use this paper’s findings to make sure that specific ECs are developed and acquired during the predicisional and preactional phases of entrepreneurial action. As the ECs in this paper are drawn from a systematic framework, policy makers and educators should ensure that ECs are developed from both entrepreneurial attitudes and personal characteristics as well as entrepreneurial motives to enhance business start-up, specifically when encouraging women to engage in entrepreneurial action (start-up).

Furthermore, this paper sheds light on how prior entrepreneurial exposure and ECs interact to impact the different action phases of the entrepreneurial process; therefore, this research is of value to the various initiatives seeking to enable women entrepreneurs through various experiential activities and EC developmental training programs.

## Limitations and Future Research Recommendations

No paper is without limitations. First, this study is conducted on women entrepreneurs in a developing country only. It will be interesting to determine if the results of this model are consistent for women entrepreneurs in developed countries. As the findings of this paper shed light on how two categories of ECs can be used to strengthen the entrepreneurial action levels of women entrepreneurs in developing countries, it will be interesting if this model will hold in developed countries in Europe and America. Furthermore, it will be fascinating to determine whether any statistically significant paths exist for prior entrepreneurial exposure with entrepreneurial action for male entrepreneurs in developed countries or whether the findings will be consistent with the findings in this paper.

Second, as statistically significant paths were only determined between prior entrepreneurial exposure and the predecisional and preactional phases, moderation effects of the ECs were not tested for the actional phase, which confirms the Rubicon crossing. Future research is needed with regards to determining the specific desires that women entrepreneurs can focus on during the actional phase of entrepreneurial action.

Third, as previous research indicated that women are more encouraged to start businesses if they were exposed to/or had women entrepreneurs as role models, it could further contribute to the prior entrepreneurial exposure construct by investigating the possibility of gender effect in a study such as this. Future studies could determine whether it makes a significant difference in women’s engagement in entrepreneurial action if the family member or role model who owns a business is female (e.g., mother or aunt) versus male.

Finally, while this paper explores five ECs, which are argued as central to the development of prior entrepreneurial exposure and action; future research could include other ECs within the entrepreneurial attitudes and personal characteristics as well as entrepreneurial motives categories that fall beyond the scope of this paper. Furthermore, certain demographic variables such as age and education could be measured to determine whether they moderate this relationship. This could be done by focusing on longitudinal and experimental designs, not only to clarify causal relationships but also to strengthen the participation of women in entrepreneurial action phases even further.

## Data Availability Statement

The datasets generated for this study are available on request to the corresponding author.

## Ethics Statement

The studies involving human participants were reviewed and approved by the Faculty of Economic and Management Sciences Ethics Committee at the University of Pretoria. The patients/participants provided their written informed consent to participate in this study.

## Author Contributions

The data were collected, and the manuscript was conceptualized and prepared by MB.

## Conflict of Interest

The author declares that the research was conducted in the absence of any commercial or financial relationships that could be construed as a potential conflict of interest.
