# Supplementary material for: Prior Entrepreneurial Exposure and Action of Women Entrepreneurs: Exploring the Moderation Effects of Entrepreneurial Competencies in a Developing Country Context
Source: Front Psychol. 2020 May 26;11:922. doi: 10.3389/fpsyg.2020.00922 (PMC7264415; doi:10.3389/fpsyg.2020.00922)
Supplement: Supplementary file 1 [file Table_1.DOCX]

**Addendum A: Moderation graphs**

To interpret the moderator effect, prior entrepreneurial exposure was split at the median to form low and high sub-groups. Then, each sub-group was plotted against the average EA 1 and EA 2, respectively. Figures 1 to 10 show the interaction effect for all the moderating variables. Therefore, interactive line graphs were used to explain the moderator effects.

**Figure 1: The moderator effect of leadership and EA 1**


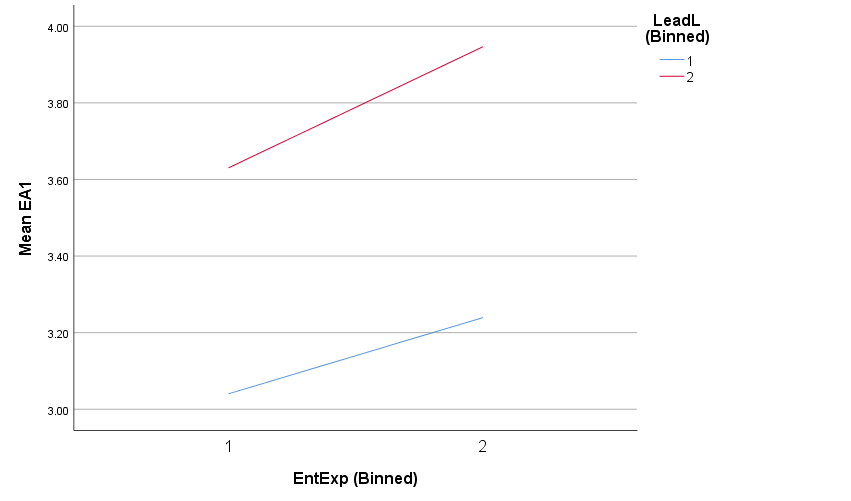


Low leadership (blue line) show that EA 1 increases less sharply as the level of prior entrepreneurial exposure increases, than for high leadership (red line) where a sharp increase in EA 1 is evident from the figure.

**Figure 2: The moderator effect of innovativeness and EA 1**


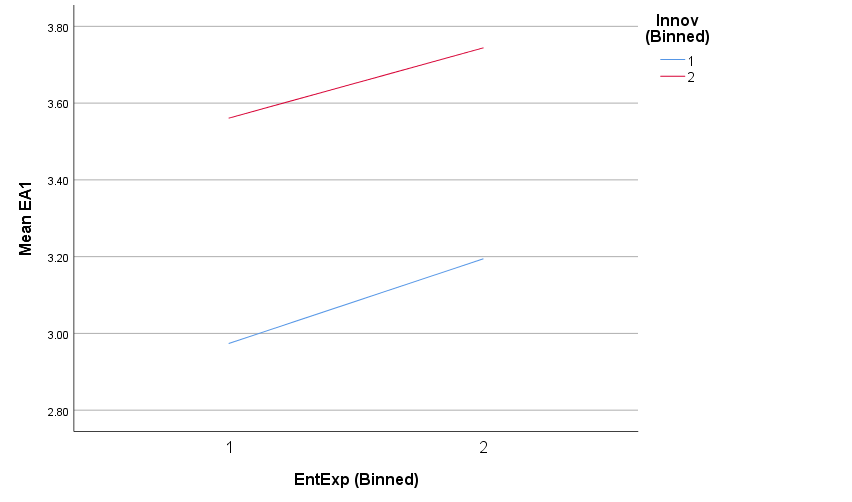


Both low innovativeness (blue line) and high innovativeness show that EA 1 increases fairly sharp as the level of prior entrepreneurial exposure increases, but at clearly differentiated levels of EA 1.

**Figure 3: The moderator effect of curiosity and EA 1**


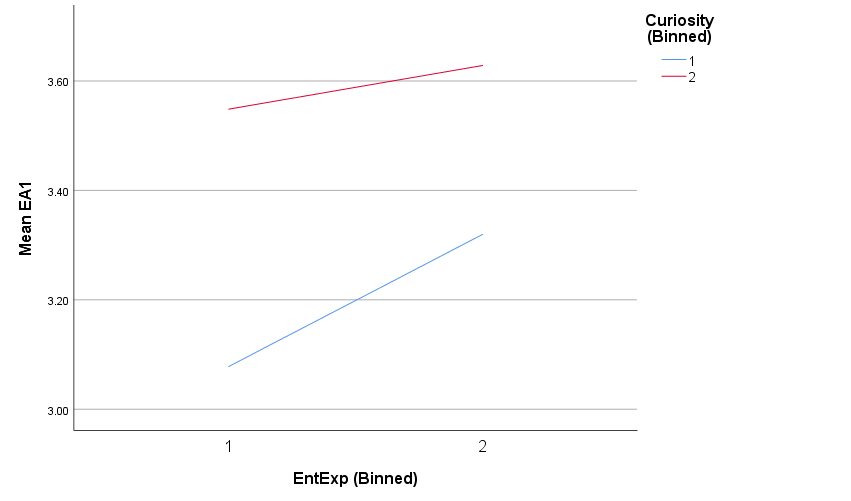


Low curiosity (blue line) show that EA 1 increases sharply as the level of prior entrepreneurial exposure increases, while for high curiosity (red line) there is only a very slight increase in EA 1.

**Figure 4: The moderator effect of self-efficacy and EA 1**


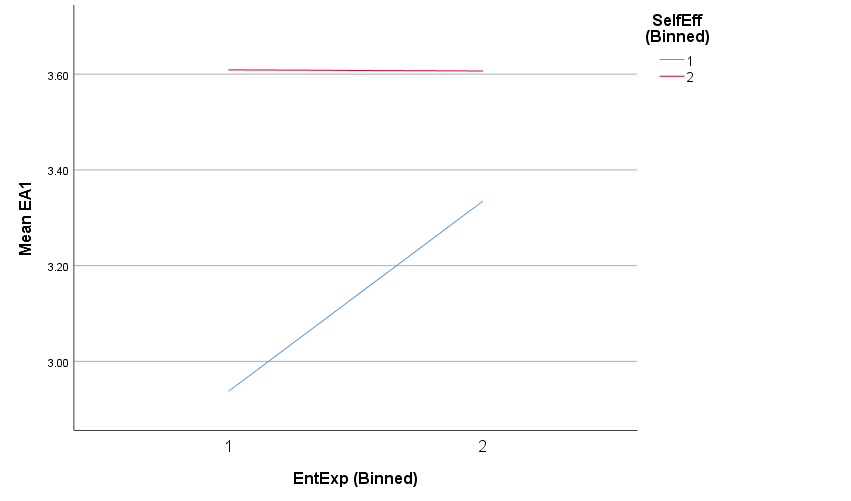


Low self-efficacy (blue line) show that EA 1 increases sharply as the level of prior entrepreneurial exposure increases, while for high self-efficacy (red line) there is basically no impact.

**Figure 5: The moderator effect of motivation and EA 1**


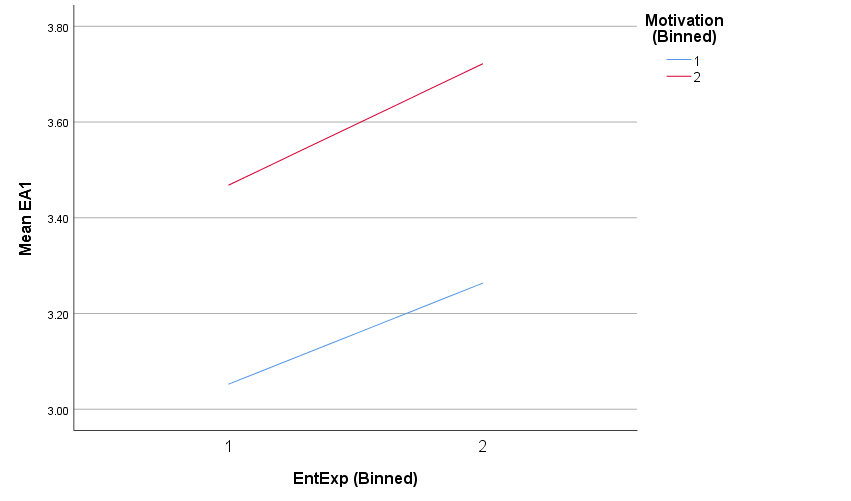


Both low motivation (blue line) and high motivation show that EA 1 increases fairly sharp as the level of prior entrepreneurial exposure increases, but at clearly differentiated levels of EA 1.

**Figure 6: The moderator effect of leadership and EA 2**


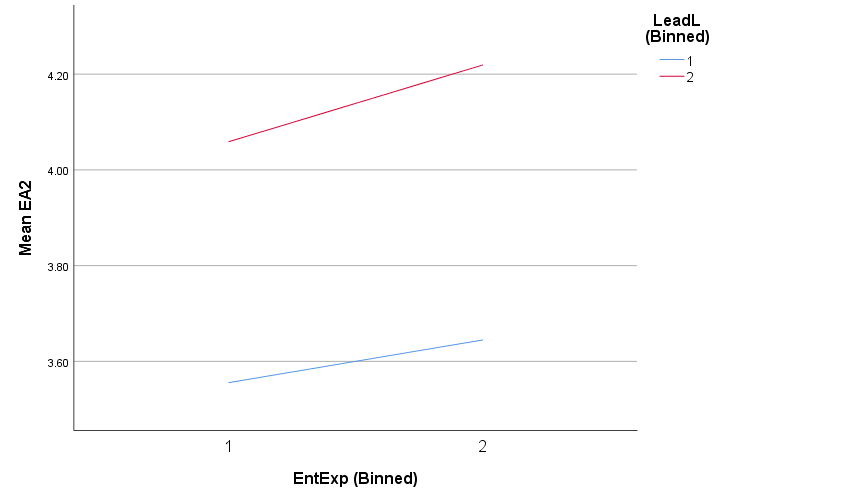


Low leadership (blue line) show that EA2 increases marginally as the level of entrepreneurial exposure increases, while for high curiosity (red line) there is a slightly more sharp increase in EA 2.

**Figure 7: The moderator effect of innovativeness and EA 2**


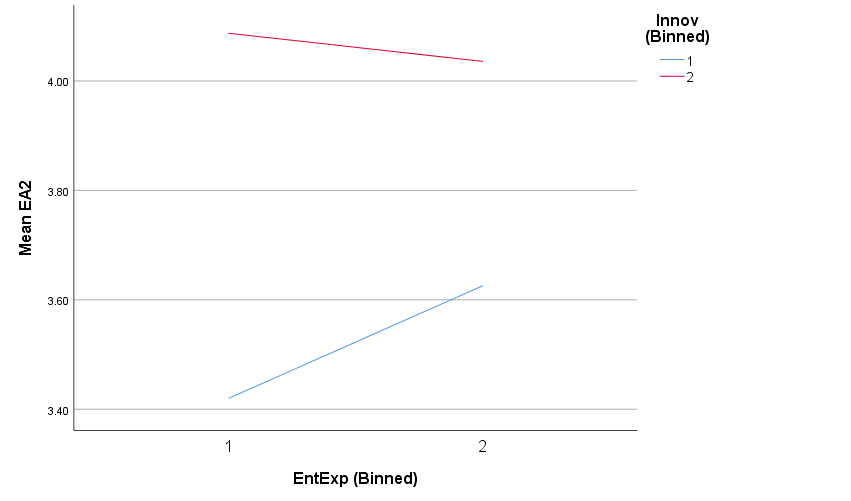


Low innovativeness (blue line) show that EA 2 increases sharply as the level of prior entrepreneurial exposure increases, while for high innovativeness (red line) there is a very marginal decrease in EA 2.

**Figure 8: The moderator effect of curiosity and EA 2**


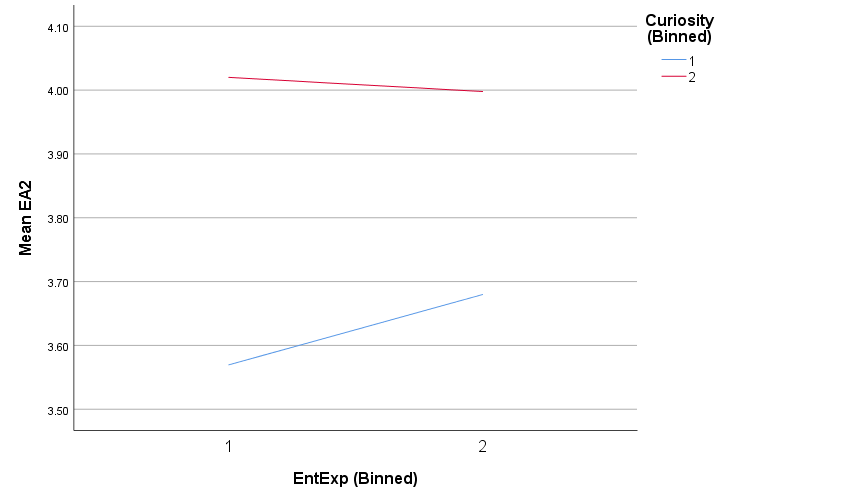


Low curiosity (blue line) show that EA 2 increases moderately as the level of prior entrepreneurial exposure increases, while for high curiosity (red line) there is a very marginal decrease in EA 2.

**Figure 9: The moderator effect of self-efficacy and EA 2**


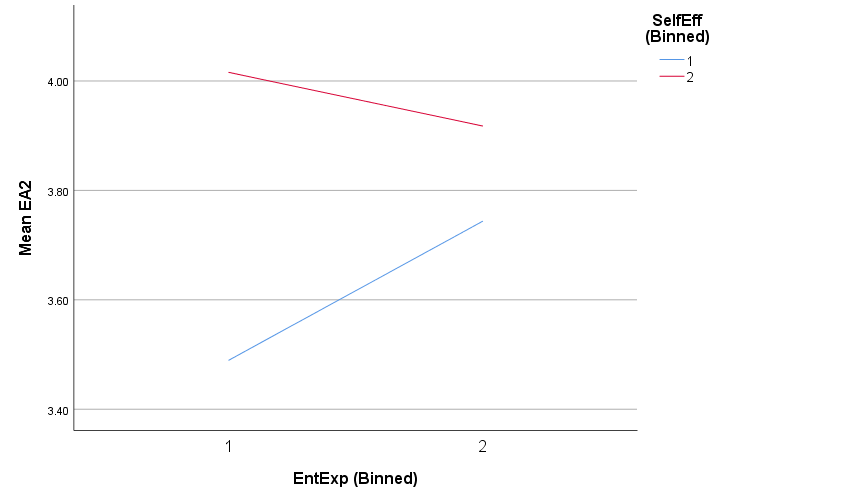


Low self-efficacy (blue line) show that EA 2 increases sharply as the level of prior entrepreneurial exposure increases, while for high curiosity (red line) there is a slightly less sharp decrease in EA 2.

**Figure 10: The moderator effect of motivation and EA 2**


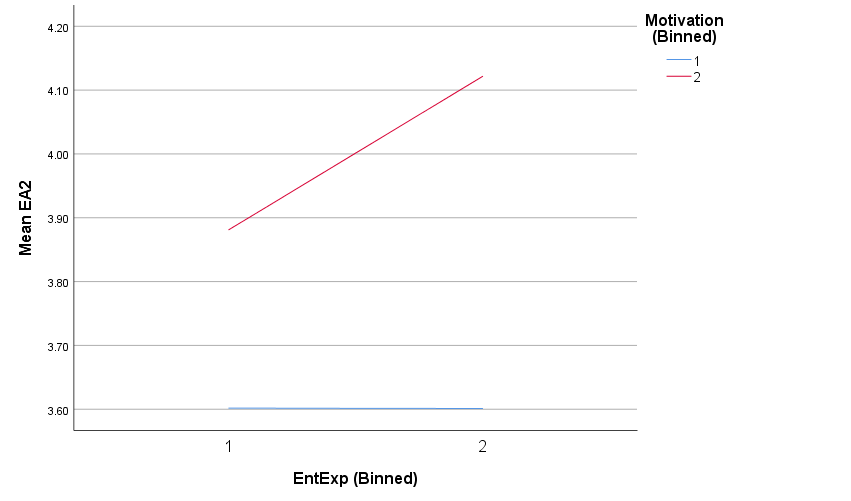


Low motivation (blue line) show that EA 2 remains at the same level, while for high motivation (red line) there is a sharp increase in EA 2.
